# Bandgap Tunable AgInS based Quantum Dots for High Contrast Cell Imaging with Enhanced Photodynamic and Antifungal Applications

**DOI:** 10.1038/s41598-018-27246-y

**Published:** 2018-06-19

**Authors:** Irshad Ahmad Mir, V. S. Radhakrishanan, Kamla Rawat, Tulika Prasad, H. B. Bohidar

**Affiliations:** 1 School of Physical Sciences, Jawaharlal Nehru University, New Delhi, India; 2 Advanced Instrument Research Facility, Jawaharlal Nehru University, New Delhi, India; 3 Special Centre for Nano Sciences, Jawaharlal Nehru University, New Delhi, India; 4 Inter University Accelerator Centre, New Delhi, India

## Abstract

Herein, we report a facile microwave-assisted synthesis of cadmium-free water-soluble silver indium sulfide (AgInS_2_ or AIS) and AgInS@ZnS (or AIS@ZnS) core-shell quantum dots (QDs) using glutathione (GSH) as stabilizer. The core and core-shell nanocrystals exhibit tunable bandgap ranging of 2.3–3.1 and 2.4–3.5 eV, mean particle size of 2.5 and 3.25 nm, quantum yield of 26% and 49%, and fluorescence lifetimes of 326 and 438 ns, respectively. The core-shell QDs exhibit color-tunable emission in the visible region (500 to 600 nm), where the tunability was achieved by varying the molar ratio of Ag:In in the precursors. *In vitro* evaluation of antifungal activity of these water/ buffer stable QDs against the fungal pathogen, *Candida albicans* demonstrated that these were not toxic to the fungal cells upto a concentration of 100 µg/ml for 16 hours of incubation. Confocal imaging and spectrofluorometric studies showed enhanced fluorescence inside the microbial cells suggesting that AIS@ZnS particles had the capability to easily penetrate the cells. The increased generation of reactive oxygen species was evaluated for the core-shell QDs (photosensitizers) by using 9, 10-anthracenediyl-bis(methylene)dimalonic acid (ABMDMA) as singlet oxygen (^1^O_2_) scavenger molecule. These QDs have the potential for use as high contrast cell imaging, photodynamic and antifungal agents.

## Introduction

Luminescent colloidal semiconductor nanocrystals, also known as quantum dots (QDs) are of great interest for their applications in numerous fields ranging from light emitting devices, photovoltaics^1^ and biomedical engineering. Besides, these are associated with versatile optoelectronic properties comprising size-dependent emission, high quantum yield (QY), good photostability and large Stokes shifts^1,2^. In contrast to conventional fluorescent probes, the narrow range of emission wavelength and high quantum yield with exceptional stability have allowed the QDs to be used in various biomedical applications such as tumor targeting, diagnostics and long term bio-imaging^3^. Among quantum dots, the II–VI materials such as CdS and CdSe are the most extensively investigated systems, mainly because of their attributes of tunable bandgap in the visible region of the spectrum, which leads to the development of high crystallinity with minor defect density and bandgap tunability^4^. Unfortunately, the commonly studied II–VI or III–V QDs, which contain “Class A metals” such as Cd, As, Hg and Pb could hardly be used in biosciences because of their known toxicity^5^. Generation of reactive oxygen species (ROS) such as hydrogen peroxide, hydroxyl radicals and singlet oxygen are well known indicators of QD cytotoxicity. Several mechanisms have been attributed to the generation of ROS by QDs. However, these results cannot be generalized for all QDs due to the variations in the synthesis protocols used and the very nature of generated QDs^6^. The toxicity of the most studied Cd-based QDs is a major drawback, limiting their biomedical applications^7^. In order to circumvent the issue of toxicity, synthesis of Cd-free QDs with improved properties and tunable emission spectra has been proposed but retaining their high brightness, narrow PL emission spectrum and high PL quantum yield identical to Cd-based QDs still remains a major challenge^8^. With advances in QD synthesis over the past few years, solution phase synthesis are used for the generation of indium phosphide (InP)^9^, copper indium sulfide (CuInS_2_)^10^, silver indium sulfide (AgInS_2_)^11^, silicon^12^ and graphene^13^ nanocrystals. These QDs hold the advantages of superior optical properties, colloidal and PL stability, and better surface chemistry over conventional Cd-based QDs. Still, there is need for synthesis of Cd-free QDs with tunable properties that can be appropriately tailored for specific applications.

Ternary group I–III–VI QDs, such as silver indium sulfide (AIS) QDs, are deemed better candidates as they contain no toxic elements and have a large absorption coefficient^14^ and longer life time^11,15–17^. AIS is a direct bandgap semiconductor with two crystal phases (chalcopyrite and orthorhombic). To date, many efforts have been made to produce AIS-based QDs. For example, hot injection technique for the synthesis of AIS QDs with photoluminescence (PL) quantum yield of 22% was reported by Chang’s group^18^. Although high-quality AIS QDs were obtained via these methods, they often required expensive and toxic organic solvents for synthesis. Additionally, the complex and harsh reaction conditions usually result in poor reproduction. Hence, it is not suitable for large-scale preparation of low-cost AIS QDs. For biological applications, hydrophobic AIS-based QDs must be transferred to the water-soluble phase via ligand-exchange or silica-coating^19^.

The use of microwave method in inorganic synthesis was first reported by Komarneni and Roy in 1985^20^. Among the 12 principles of green chemistry “safer solvent and energy efficiency” are the two key principles for synthesis of nanomaterials^21^. American Chemical Society monograph on Green Chemistry recommends the method that requires minimum energy for a reaction to take place^22^. Therefore, microwave assisted method is a green approach for nanomaterial and quantum dots synthesis and offers the advantages of rapid synthesis, low power consumption, reproducibility and uniform heating^20–27^. Thus, the green, microwave assisted aqueous synthesis of AIS-based QDs was undertaken in this study as an effective alternative to the conventional synthesis method.

Nanotechnology provides a new platform to engineer different materials at nanoscale with desired physicochemical properties and harness them for biological applications^28^. The use of nanotechnology in medicine is a recent trend and complements the search for new antimicrobial agents and alternatives, which has gained impetus due to the rising threat to global public health posed by multidrug resistance (MDR) in microbial pathogens^28,29^. Rampant use of antibiotics has led to the increasing incidence of MDR in microbial flora. Various recent reports demonstrate the antimicrobial potential of nanomaterials and their combinations with existing drugs^28^. In this report, we have developed a green and facile aqueous route for the synthesis of highly luminescent AIS@ZnS QDs through microwave irradiation using GSH (glutathione) as capping agent. These Cd-free QDs exhibit tunable bandgap by adjusting the Ag:In molar ratio in the precursor. The antifungal activity of the Cd-free QDs was studied against the opportunistic fungal pathogen, *Candida albicans*. *Candida* is a dimorphic, commensal organism present in the natural microbiota of every individual, which turns pathogenic under immunocompromised conditions to cause fungal infection, *Candidiasis*. *Candida* spp. are major fungal pathogens which cause both superficial and life threatening systemic mycoses. Higher incidence of fungal infections, emergence of drug resistant fungal isolates and limited availability of antifungal drugs are the leading causes for the significant rise in the mortality and morbidity in imunocompromised individuals and patients with serious diseases^29^. *Candida* is used, in our case, as a model microorganism representing fungal pathogens for the study of antifungal property of synthesized QDs and the results will also be relevant for other clinically important fungal pathogens.

Cellular uptake of QDs are essential parameters for establishing the role of QDs as antimicrobials at toxic concentrations or as biostable fluorescent markers at sub-inhibitory concentrations^30^. Studies have assessed the cellular uptake of Cd-based QDs in human cell lines^30^. The surface modifications of QDs facilitate their internalization into the cells and are critical determinants for enhancing their cellular uptake. Hence, for biological and medical applications, the cellular uptake and toxicity of the QDs need to be evaluated. In this study, we analyzed the cellular uptake of the Cd-free QDs by the *Candida* cells and determined their toxic concentration for the fungal cells. The cellular uptake and subsequent toxicity of QDs have been linked to the augmentation of intracellular ROS. Therefore, optimization of QDs in terms of minimizing oxidative stress in the cells is imperative for their realistic nano-bio applications. Herein, we assessed the intracellular ROS to understand the oxidative stress induced post internalization of the Cd-free QDs in the fungal cells, to establish their potential capability at sub-inhibitory concentrations as bioimaging and drug delivery agents.

QDs are known to be capable of directly photosensitizing the production of singlet oxygen (^1^O_2_)^31^. Photodynamic therapy (PDT), a non-invasive therapeutic strategy for cancer is based on the generation of singlet oxygen (^1^O_2_) and involves photosensitizer (PS) drugs and external light for therapy. Conventional PS drugs mediated PDT still has to face challenges because of poor water solubility and lack of tissue specificity. However, efforts are in progress to develop nano-platforms to enhance the efficacy and tumor selectivity of PDT. In this study, we evaluated the photo-stability and photodynamic efficiency of the synthesized Cd-free, AIS-based ternary QDs for assessing their potential in delivery of photosensitizers for cancer diagnosis and photodynamic therapy (PDT) applications.

## Experimental Section

### Materials

Indium (III) nitrate hydrate (In(NO_3_)_3_ ·xH_2_O, 99.99%), glutathione (GSH, 98%), silver nitrate (AgNO_3_, 99%), sodium sulfide nanohydrate (Na_2_S·9H_2_O, 99.99%), zinc acetate dehydrate (Zn(Ac)_2_·2H_2_O, 98%) and 9, 10-anthracenediyl-bis (methylene) dimalonic acid (ABMDMA) were purchased from Sigma Aldrich (USA). Rhodamine 6G (99%) was purchased from CDH, India. All these were used as received.

Media chemicals (Yeast extract, Peptone, Glucose and Agar) for culture of the microbial cells were procured from HI-Media (Mumbai, India) and Fisher Scientific (Mumbai, India). Analytical grade Sodium chloride, Potassium hydroxide, Disodium hydrogen orthophosphate and Sodium dihydrogen phosphate obtained from Qualigens, India were used for preparing buffers. Fluorescent probe 2, 7, DichlorofluorescinDiacetate (DCFDA) was obtained from Sigma (USA). All the procedures were performed at room temperature 25 °C, unless synthesis otherwise stated.

### Synthesis of AIS@ZnS QDs

In a typical synthesis, 0.02 mmol of Ag(NO_3_), 0.08 mmol of In(NO_3_)_3_ ·xH_2_O, and 0.4 mmol of GSH was added to 42 ml of deionized water in a 200 ml capacity vessel. The solution color changed from turbid to clear under vigorous stirring when pH was adjusted to 8.5 using 1.0 M NaOH solution. Then, a freshly prepared Na_2_S solution (0.05 M) was added to the mixture solution and then this solution was exposed to microwave radiation (at 160 W) for 5:30 min. The production of AIS QDs was inferred from the appearance of a clear golden color of the dispersion. The hot reacted mixture was allowed to cool to a temperature less than 50 °C. Again 0.1 M of Zn(Ac)_2_ and 0.05 M of Na_2_S solutions were added for the preparation of the core-shell structures. This preparation was further irradiated at 160 W for 5:30 min which produced AIS@ZnS core-shell structures.

For the detection of singlet oxygen by photobleaching of ABMDMA, we prepared solution of ABMDMA in water at a concentration of 5 mg/ ml (12 mM) of water. We further diluted this solution to 24 µM immediately before use.

### Strains and Growth media

Wild type SC5314 *Candida albicans* strain^32^ was used for this study and strain description is reported elsewhere. The fungal strain was maintained in liquid YEPD broth containing 1% (w/v) Yeast extract, 2% (w/v) Dextrose and 2% (w/v) Peptone and 2.5% (w/v) Agar was added to the Petri plates for solid media.

Strains were preserved in 15% (v/v) glycerol at −80 °C. Cells were revived on YEPD plates at 30 °C from frozen glycerol stocks and maintained at 4 °C. Liquid media cell cultures were grown at 30 °C with continuous shaking at 140–150 rpm for 14–16 hrs and these exponentially growing cells were used for all the experiments.

## Experimental Methods

### Characterization Techniques

Transmission electron microscopy (TEM) images (150,000X) were obtained on a JEOL 2100F TEM operating at a voltage of 200 kV. For more details see refs^33–35^. The zeta potential measurement was carried out on an electrophoresis instrument (ZC-2000, Microtec, Japan)^33,34^. X-Ray diffraction (XRD) patterns were obtained on a XRD, Rigaku D/Max 2200 diffractometer with CuKα radiation (λ = 1.5406 Å) in the 2θ range of 10–70°^33–35^. UV-Vis absorption spectra and steady state fluorescence (FL) spectra were recorded on Cecil model CE-7200 (Cecil Instrument, UK) spectrophotometer^33,34^ and Varian Cary Eclipse Fluorescence spectrophotometer^34,35^, respectively. Fluorescence lifetime measurements were carried out using a frequency-doubled Ti:Sapphire laser (Coherent Mira) and Silicon avalanche photodiode (Multi-Photon Devices PDM) detector. Dynamic light scattering measurements was prepared to determine the size distribution histogram of the QDs in dispersion. A 1024-channel correlator (PhotoCor USA) was used for this purpose.

### Assessment of susceptibility of *Candida* cells to Quantum Dots

Broth micro dilution assay was used to determine the minimum inhibitory concentration (MIC) of the *Candida* cells towards the Quantum dots (AIS and AIS@ZnS). MIC was determined by the broth micro-dilution method^36,37^ as per the recommendations of the Clinical and Laboratory Standard Institute (CLSI). Briefly cells were grown for 14–16 hrs on agar plates (exponentially growing log phase cells) and re-suspended in 0.9% saline to give an optical density of 0.1 at 600 nm (OD_600_), which corresponds to cell number 0.5–1 × 10^6^ cells**/** ml. Further the cells were diluted 100-fold in YEPD medium to make the final concentration of cells 0.5–1 × 10^4^ cells/ ml. The cells were allowed to grow at different concentration of the two Quantum Dots AIS and AIS@ZnS at 30 °C with continuous shaking. In this method, growth of the cells were evaluated both visually and by taking OD_600nm_ in a microplate reader. Readings were recorded at 600 nm after 48 hrs and growth differences of the fungal cells grown in the presence of QDs were evaluated comparing with control (no QDs used).

### Cellular Uptake of Quantum Dots by the *Candida* cells

Cellular uptake of Quantum dots (AIS and AIS@ZnS) was assessed to check the internalization of the QDs in the fungal cells according to a previously described protocol with slight modifications^30^. Cells grown till mid exponential phase were harvested, followed by washing with PBS buffer pH 7.4 twice to remove the media. Then, cells were re-suspended in PBS pH 7.4 at a concentration of 10^7^ cells/ml and incubated separately with AIS (31.25 µg/ml) and AIS@ZnS (62.5 µg/ml). From each of these two sets, 10^7^ cells were taken out at various time points viz. 10 min, 20 min, 30 min, 60 min, 2 hrs, 4 hrs, 6 hrs, 8 hrs, 10 hrs, 12 hrs, 14 hrs, 16 hrs, 24 hrs and 48 hrs. The aliquot of cells taken out were harvested and the supernatant was removed. The washed cells were resuspended in 1 ml PBS and relative fluorescence Intensity (*Rf*) was measured in a Perkin Elmer L55 spectrofluorimeter at respective excitation and emission wavelengths of 460 and 600 nm with slit widths of 10 nm, for AIS and respective excitation and emission wavelengths of 425 and 575 nm with slit widths of 10 nm, for AIS@ZnS. The blank (absence of QD) was maintained separately to avoid auto-fluorescence. To confirm the presence of QDs within the cells, the images of the fluorescence within the cells were taken in the confocal microscope *FluoView™ FV1000*.

### Measurement of intracellular Reactive Oxygen Species (ROS)

Intracellular ROS generated has been detected using a fluorogenic cell permeant dye DCFH-DA in microbial pathogens which include *Leishmania donovani*^38–40^, *Candida albicans*^41^, *Saccharomyces cervisiae*^42^, *E. coli*^43,44^. Intracellular ROS levels in response to QDs were measured in this study as reported previously with slight modifications^41^. Cells were grown till mid exponential phase in the presence of AIS (31.25 and 62.5 µg/ml), AIS@ZnS (62.5 and 125 µg/ml) and absence of QDs (growth control). Cells were then harvested, followed by washing with PBS buffer pH 7.4 twice to remove the media. 10^7^ cells were re-suspended in 1 ml PBS pH 7.4. The fluorescent probe DCFH-DA (final concentration 10 µM) was added to each cell suspension and incubated at 30 °C for 1 hr. Relative fluorescence Intensity (Rf) was measured in a Perkin Elmer L55 spectrofluorimeter at respective excitation and emission wavelengths of 485 nm and 530 nm with slit widths of 10 nm. The blank (absence of QDs) was maintained separately to avoid auto-fluorescence. To confirm the presence of ROS, the images of the fluorescent cells were taken in the confocal microscope *FluoView™ FV1000*.

## Results and Discussion

### Structural characterization

**Figure 1:**
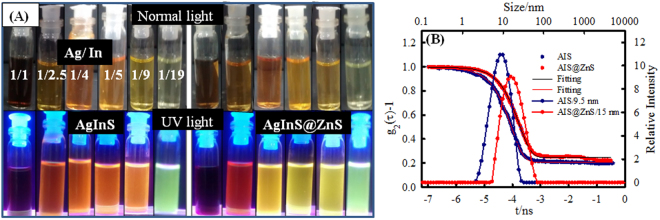
(**A**) Images of AIS and AIS@ZnS core-shell quantum dots in day light and under UV radiation. For core and core-shell structures, photos depict the coloration as function of Ag:In molar ratio (1:1, 1:2.5, 1:4, 1:5, 1:9, 1:19). It is clearly seen that the color change under UV illumination indicates the fluorescent nature of the quantum dots (left panel). (**B**) Shows correlation relaxation decay and size distribution of AIS and AIS@ZnS core-shell quantum dots.

**Figure 2:**
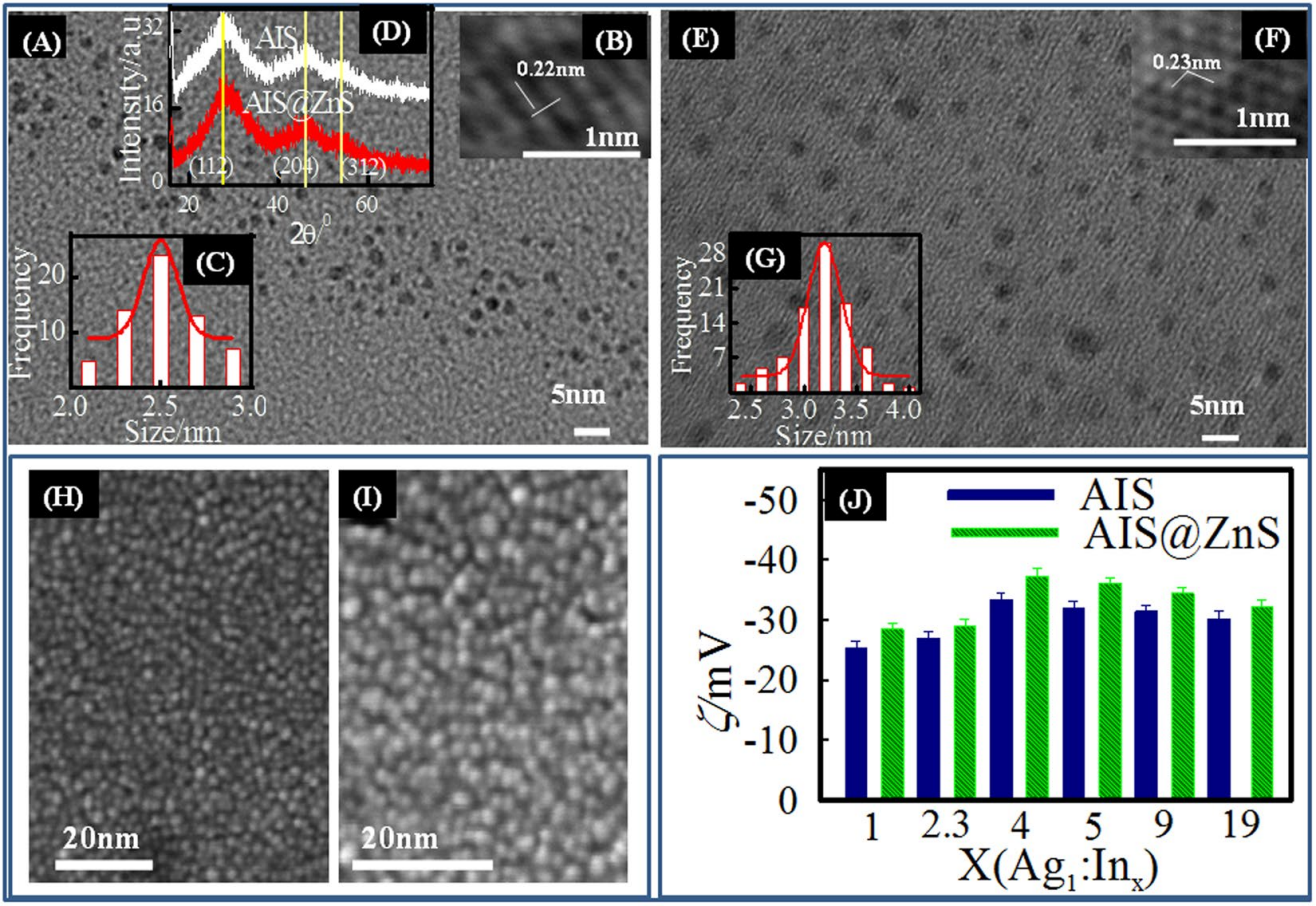
(**A**) TEM, (**B**) HRTEM images and (**C**) size histogram of AIS core only. (**D**) XRD of AIS core and AIS@ZnS core-shell. (**E**) TEM, (**F**) HRTEM images and (**G**) size histogram of core-shell QDs. These images and XRD are for 1:4 (Ag:In) ratio samples. (**H** and **I**) are FESEM images of AIS and AIS@ZnS QDs, respectively. (**J**) Zeta potential distribution plot of core and core-shell quantum dots with different ratio of Ag:In.

The synthesized AIS based core-shell QDs exhibited high luminescence that could be visualized directly under UV light (Fig. 1). After the visualization of luminescence, it was important to know the particle size, structure and crystallinity of synthesized QDs. Figure 2A shows the monodispersed AIS QDs with average size of about 2.5 nm, and clear lattice fringes. After ZnS coating, the average size of the QDs (core-shell) structures increased to 3.25 nm (Fig. 2E). HRTEM (Fig. 2B and F) of AIS and AIS@ZnS QDs was used to characterize their crystalline structure. The spacing between crystal planes was found to be 0.22 and 0.23 nm, respectively for AIS and AIS@ZnS QDs. FE-SEM images (Fig. 2H and I) of AIS QDs and AIS@ZnS QDs depict uniform spherical structures of these quantum dots.

The size distribution of the QDs in their dispersion state is shown in Fig. 1. The mean size determined from the measured intensity correlation function g_2_(τ) was 9.5 nm for core-only and 15 nm for core-shell structures. The higher size observed (compared to the TEM data) can be attributed to hydration driven oligomerization of the QDs which is not unusual in colloidal dispersions.

### Electrophoresis studies

It was found that the core-only QDs showed negative zeta potential (Fig. 2J) while the core-shell QDs had marginally higher zeta potential of same polarity. Zeta potential measurements showed highest potential of ≈ −40 mV for core-shell samples made with precursor composition of 1:4 (Ag:In) molar ratio samples, indicating highest stability. In colloid science, it is well known that particles with zeta potential ≥ ± 30 mV offer the best dispersion with long term dispersion stability.

### Crystalline morphology

**Figure 3:**
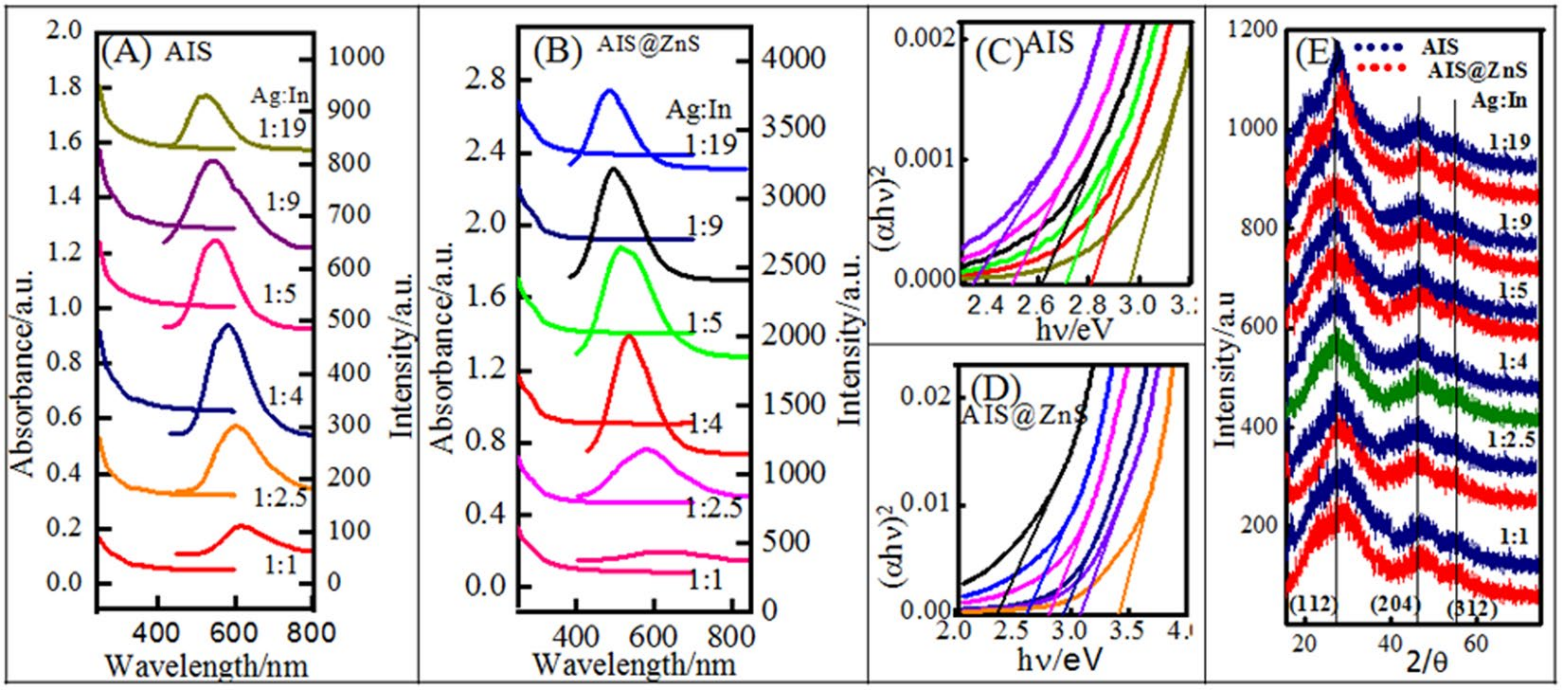
(**A**) Absorbance, (**B**) fluorescence spectra, (**C**) and (**D**) Tuc plot (Bandgap plot) and (**E**) XRD profiles of AIS core and AIS@ZnS core-shell QDs shown for variable Ag:In ratio.

The crystalline morphology of the as-prepared QDs were determined by subjecting the samples to X-ray diffraction (XRD) studies and the data exhibited three broad peaks located at 2θ ≈ 27.2°, 45.4° and 54.3° representing (112), (204) and (312) diffraction planes, respectively. Such assignment corresponds to the tetragonal AIS crystal (JCPDS NO32–0483) (Fig. 3E). Similar diffraction peaks were also observed from AIS@ZnS samples, but here peaks were marginally shifted towards higher 2θ values with respect to the same of AIS QDs. These facts indicated that the obtained QDs were not a mixture of ZnS and AIS, but a certain amount of Zn^2+^ ions was introduced which had diffused into the nanocrystal structure of AIS. This could be responsible for the slight difference in the XRD pattern compared to that of AIS samples^45^.

XRD profiles and TEM images of the samples made with different Ag:In ratios is presented in Figs 3E and S1 (Supplementary Information). These results clearly indicated that the crystal structure and particle size remained invariant of Ag:In ratio, barring the 1:1 (Ag:In) sample which showed the size of 5.5 and 8 nm respectively for AIS and AIS@ZnS with very low QY of 0.8% (Fig. S3B (Supplementary Information)).

### Spectroscopic studies

**Figure 4:**
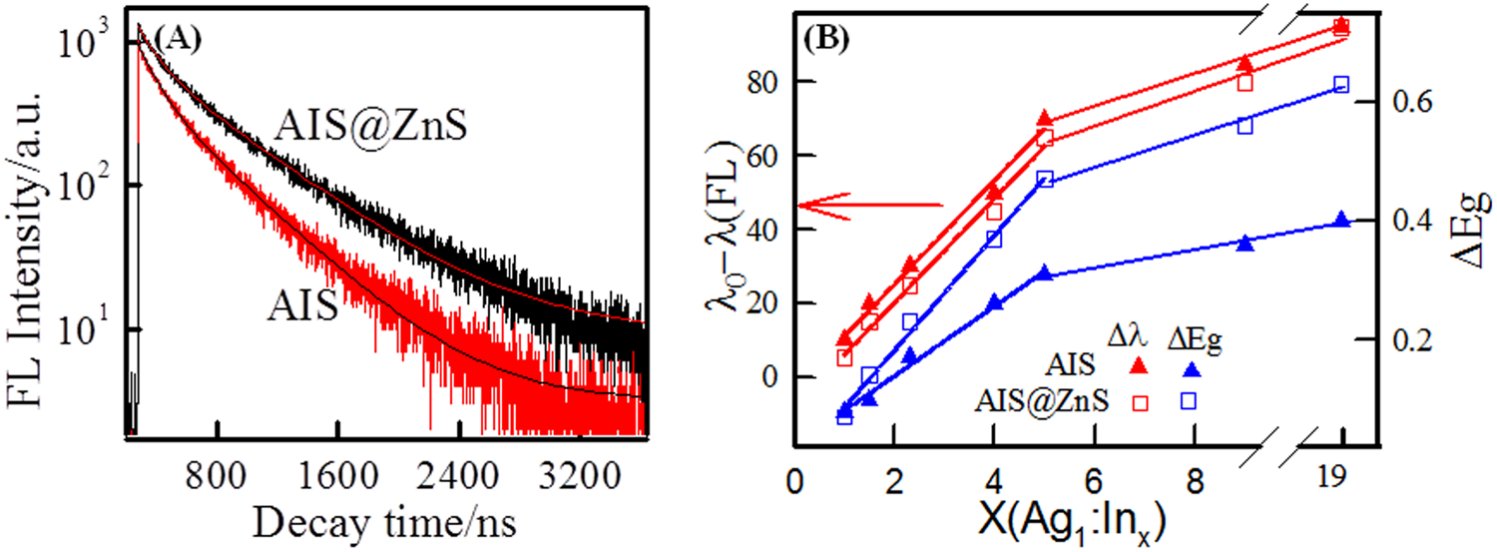
(**A**) FL decay curves of AIS and AIS@ZnS quantum dots. (**B**) Dependence of shift in wavelength and bandgap with changing cation ratio with respect to Ag:In 1:1 (minimum quantum yield) sample.

The absorbance and fluorescence (FL) spectra of AIS and AIS@ZnS QDs are shown in the Fig. 3A and B, respectively. As seen from Figs 3 and S2I and J (Supplementary Information) emission spectra of AIS and AIS@ZnS QDs show Stokes shift of about 100 nm as function of Ag:In ratio. Also it is notable that FL emission of AIS@ZnS QDs shows considerable blue shift compared to AIS. Interestingly, these quantum dots displayed emission in the visible region (550–650) with varying Ag:In ratio. In addition, these QDs exhibited quantum yields in the range of 0.8–26% and 2–49%, respectively for core and core-shell QDs, with change in Ag:In molar ratios from 1:1 to 1:19. Figure 4B shows the linear dependence of shift in emission wavelength and bandgap with change in Ag:In ratio (1:1 to 1:5). This linear dependence disappeared on increasing the Indium content (see Fig. S2 (Supplementary Information)). Interestingly, even though the size of these QDs (Figs 2 and S1 (Supplementary Information)) do not show any significant change, their bandgaps (Fig. 3C and D) show considerable changes in the range of 2.3–3.1 and 2.4–3.5 eV, respectively for AIS and AIS@ZnS QDs with change in Ag:In ratio.

For the study of FL properties of these QDs, the optimization was done as a function of pH value (Fig. S2A and B (Supplementary Information)) in the range between 8 to 11. We noticed that when pH was below 8, the solution was turbid. When we adjusted the pH value to 8.5, the FL intensity reached its maximum. The investigation of In:GSH molar ratio dependence (Fig. S2C and D (Supplementary Information)) showed a significant effect on the quantum yield of these nanocrystals. The maximum FL intensity was obtained corresponding to the In:GSH ratio of 1:5. Besides this optimization, we also investigated the effect of duration of microwave irradiation (Fig. S2E and F (Supplementary Information)) and the In:S molar ratio. Fig. S2G and H (Supplementary Information) showed variation in the QY values. The samples ware irradiated for 5 min and 30 sec (5:30 min), that was the optimized irradiation time, and In:S molar ratio was 2:1 which yielded the highest QY (see Fig. S3 (Supplementary Information)). The quantum yields (QY) were determined following the procedure outlined in refs^33,34^.

### Elemental identification

The composition analysis and elemental identification were determined from the SEM/ EDX spectra shown in Fig. S4 (Supplementary Information) and the analyzed results are shown in Table S1 (Supplementary Information). Here EDX analysis shows the phase purity and presence of Ag, In and S in core only and Ag, In, S and Zn in core-shell QDs.

### Fluorescence Lifetime

The Fluorescence decay curves are shown in Fig. 4A and their lifetime data are listed in Table S2 (Supplementary Information). In general, the relaxation profile did not follow single exponential decay, but a bi-exponential decay function could be easily fitted to the data. The decay time was decomposed into fast τ_1_ and slow τ_2_ mode decay components using the following equation,

1 $${\boldsymbol{F}}({\boldsymbol{t}}){\boldsymbol{=}}{{\boldsymbol{A}}}_{{\boldsymbol{1}}}\exp {\boldsymbol{(}}{\boldsymbol{-}}t{\boldsymbol{/}}{{\boldsymbol{\tau }}}_{{\boldsymbol{1}}}{\boldsymbol{)}}{\boldsymbol{+}}{{\boldsymbol{A}}}_{{\boldsymbol{2}}}\exp {\boldsymbol{(}}{\boldsymbol{-}}t{\boldsymbol{/}}{{\boldsymbol{\tau }}}_{{\boldsymbol{2}}}{\boldsymbol{)}}$$

where, A_1_ and A_2_ represent the weights of the decay components τ_1_ and τ_2_ at t = 0. The fast decay component τ_1_ was due to intrinsic recombination processes of initially populated core states and surface defect states. The slow decay time τ_2_ was interpreted as arising from radiative recombinations of donor-acceptor pairs or their deep defect recombination mechanisms^13^. The average decay time determined was 438 ns for core-shell and 326 ns for core QDs. It is to be noted that coating of ZnS shell over AIS-core increased its lifetime as compared to core-only structures. This indicated that some of the surface-related non-radiative centers were eliminated by ZnS layers. It was also noticed that the lifetime of core-shell structures was much longer than that of most organic dyes, carbon QDs and some II–VI QDs^11,12,16,17,46^. Thus, our core-shell luminescent QDs can be used for fluorescence based, time lapse live cell imaging applications to continuously monitor living specimens over extended periods of time. Table 1 shows the comparisons of quantum yield and lifetime AIS based core-shell QDs synthesized via different methods.

**Table 1:** Comparison of QY and FL life time of AIS based core-shell QDs.

Method	PL%	Life time/ns	Ref.
Conventional heating	20	124–170	^15^
Electric pressure cooker	39	243	^11^
Hot injection method	15	286 to 984	^16^
Hot injection method	32	552	^17^
thermal decomposition	41	79–441	^45^
Organic Phase	23	120–145	^46^
Organic Phase	50	140–240	^36,51^
Microwave	49	438	Present work

### Detection Singlet oxygen by photobleaching

Photodynamic therapy (PDT) is mostly based on the generation of singlet oxygen (^1^O_2_) through complex interaction of photosensitizer, light and oxygen (^3^O_2_). The water soluble 9, 10- anthracenediylbis(methylene)dimalonic acid (ABMDMA) was used as singlet oxygen (^1^O_2_) scavenger (trap) molecule. The ^1^O_2_ output of the both AIS and AIS@ZnS QDs were studied in aqueous solutions. The scavenger molecule is a derivative of anthracene that reacts with ^1^O_2_ to produce endoperoxide, which in turn decreases the ABMDMA absorption.

**Figure 5:**
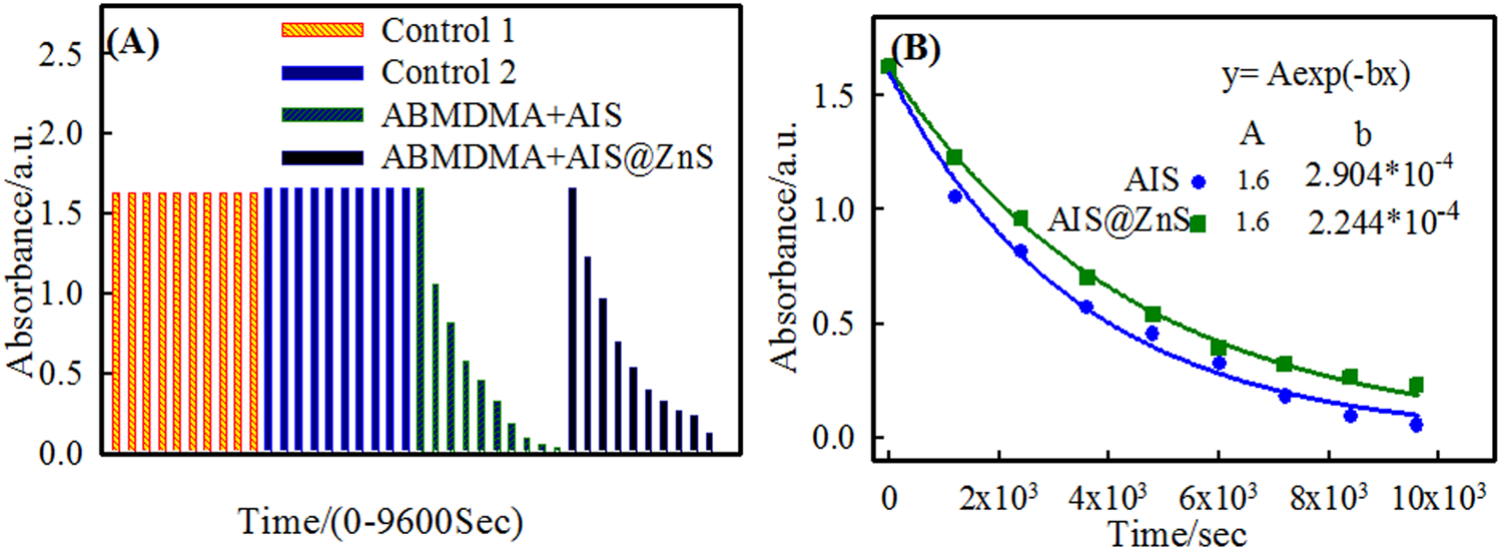
(**A**) Time dependent changes in optical density at 400 nm of ABMDMA irradiated (control 1), ABMDMA-QD without irradiation (control 2), ABMDMA-AIS irradiated and ABMDMA-AIS@ZnS irradiated after the time periods (0, 20, 40, 60, 80, 100, 120, 140 and 160 min). (**B**) Shows the exponential decay fitted curve with respect to time.

This reaction was monitored using a spectrophotometer by observing the time dependent decrease in absorbance at 400 nm (λ_max_) which is shown in Figs 5 and S5 (Supplementary Information). In a typical experiment 24 µM of ABMDMA (in water) was mixed with QDs for irradiation by 8 W UV lamp delivering a probe density of 280 μw/cm^2^. The light dosage used were 336, 672, 1008, 1344, 1680, 2016 and 2352 mJ.

These solutions were irradiated in a quartz cuvette. The absorbance spectrum was recorded after every 20 min of irradiation which is shown in Fig. S5 (Supplementary Information). For the control we did two experiments, measuring the absorbance (of (i) 24 µM of ABMDMA in water followed by irradiation and (ii) 24 µM ABMDMA in presence of (0.1 mg) QDs without irradiation), after every 20 min as is shown in Fig. 5. Under these conditions the UV-Vis spectrum of ABMDMA remained unchanged with time. On the other hand, we did the same experiments of ABMDMA in presence of (0.1 mg) AIS and AIS@ZnS followed by irradiation, which showed the time dependent decrease in the optical density of ABMDMA. These measurements led to a 97% and 95% decrease (Figs 5 and S5 (Supplementary Information)) in the ABMDMA-AIS and ABMDMA-AIS@ZnS spectra respectively, after 160 min. This confirmed the production of singlet oxygen under illumination conditions. Also, we fitted the absorbance data A(t) presented in Fig. 5B to a single exponential decay function given by

2 $${\boldsymbol{A}}({\boldsymbol{t}}){\boldsymbol{=}}{{\boldsymbol{A}}}_{{\boldsymbol{0}}}{{\boldsymbol{e}}}^{{\boldsymbol{-}}\mathrm{kt}}$$

where, A_0_ is a constant and k is quenching rate constant.

From the fitted data, we find the value of quenching rate constant k as 3.44 × 10^3^ and 4.46 × 10^3^ s^−1^, respectively for AIS-ABMDMA and AIS@ZnS-ABMDMA mixtures that were illuminated under UV light.

### Cytotoxicity

**Figure 6:**
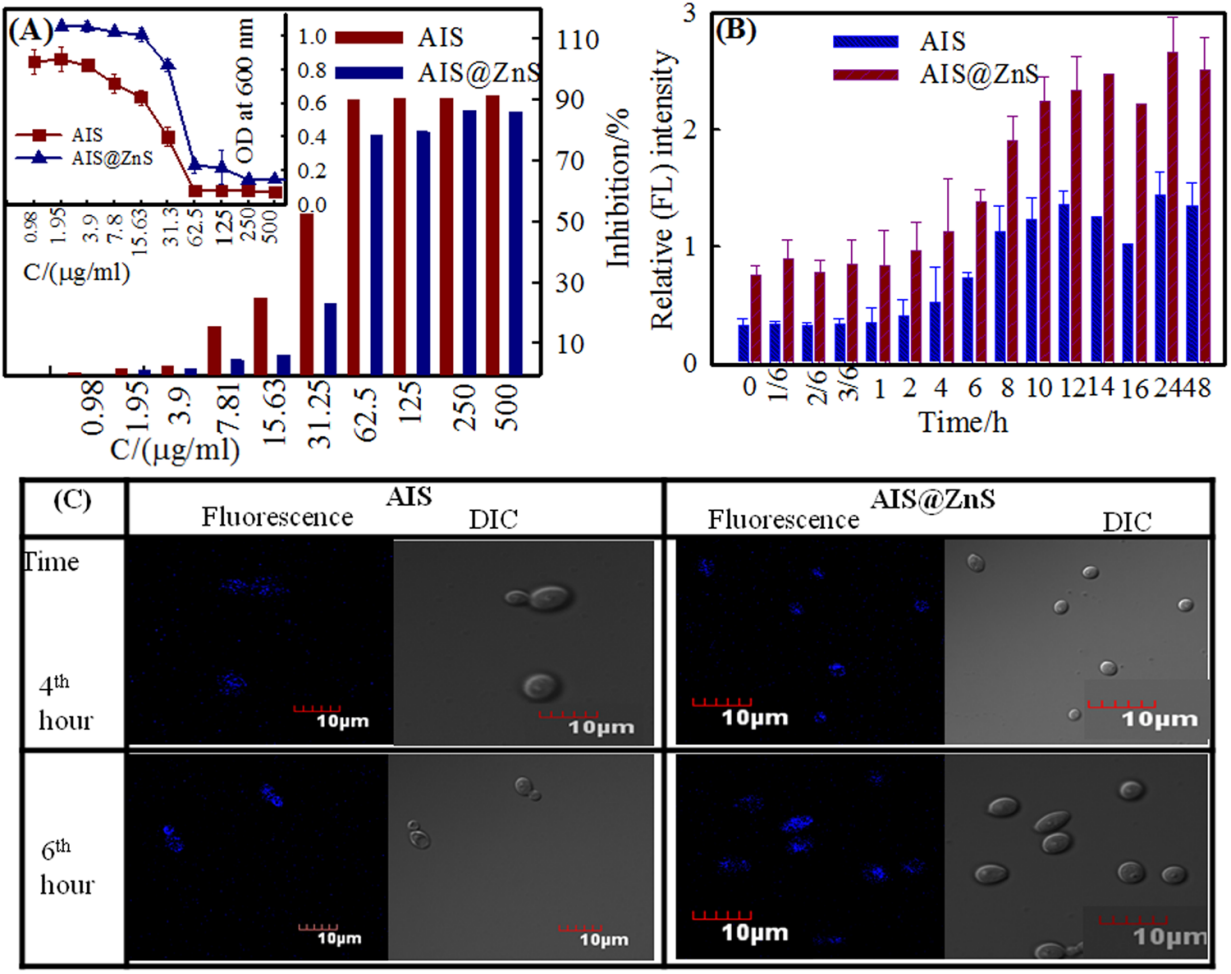
(**A**) Antifungal activity of QDs determined by broth microdilution method for *Candida* cells grown in the absence (Control) and presence of AIS and AIS@ZnS QDs respectively showed dose dependent percentage inhibition of growth for *Candida* cells. (Inset) Determination of OD_600nm_ after 48 hrs for *Candida* cells grown at 30 °C in presence of AIS and AIS@ZnS QDs respectively. (**B**) Cellular uptake of AIS and AIS@ZnS QDs is estimated by relative fluorescence (Rf) intensity values and Y axis depict the mean Rf values ± the standard deviation (SD) of three sets of independent experiments. Rf Intensity was calculated by subtracting the fluorescence intensity for cells incubated without QDs (blank) from fluorescence intensity for cells incubated with AIS and AIS@ZnS QDs respectively. (**C**) Confocal images showing cellular uptake of QDs after incubation for 4 and 6 hours. The blue fluorescence inside the fungal cells showed the internalization of the QDs.

This experiment was carried out to find if surface modified QDs could be used as biostable fluorescent markers for live imaging in microbial cells. The cellular toxicity of the two surface modified QDs was determined by broth microdilution assay against the common fungal pathogen, *Candida albicans*. Individual effects of different concentrations of QDs was investigated against this pathogen (Fig. 6). Broth microdilution assay revealed that minimum inhibitory concentration required to inhibit 90% of organisms (MIC_90_) for AIS was 62.5 µg/ml whereas for AIS@ZnS was 125 µg/ml (Fig. 6A). Figure 6A clearly shows the QD concentration dependent percentage inhibition of growth of fungal cells and therefore, can be used to kill the microbes at the above mentioned respective concentrations of AIS and AIS@ZnS. Surface modifications may be the likely reason for the reduction in the cytotoxicity of QDs against microbial cells. The lower toxicity of QDs for microbial cells is most likely to make the application of QDs desirable for any kind of biological imaging at sub-inhibitory concentration. The toxicity and uptake of QDs are both dependent on their physicochemical properties.

### Cellular uptake

Uptake of QDs is important for assessing their internalization within the microbial cells. Cellular uptake of both the QDs was determined at sub-inhibitory concentrations of AIS (31.25 µg/ml) and AIS@ZnS (62.5 µg/ml) respectively in order to be able to predict the possibility of use of QDs for live imaging in microbial cells and other biological applications. Figure 6B and C shows the quantitation of cellular uptake in terms of fluorescence intensity. Our results clearly indicate that there was higher uptake of AIS@ZnS as compared to AIS at all the time points examined, which was evident from the higher fluorescence intensity exhibited by AIS@ZnS sample (Fig. 6B). We found that the observed difference in the fluorescence intensity for the internalized QDs (AIS and AIS@ZnS) shown in Fig. 6B and C corroborated with the cellular uptake quantified for the respective QDs. The blue fluorescence inside the fungal cells in the confocal images shows the internalized QDs (AIS and AIS@ZnS) in fungal cells after incubation for 4 and 6 hrs (Fig. 6C). The *Candida* cells attain exponentially growing logarithmic phase between 8 to 14 hours and surprisingly, the uptake of the QDs also appeared to be the maximum between these time intervals (when the cells are in their highest metabolic state), attaining saturation beyond the log phase of growth.

The degree of quantum dot toxicity has not yet been extensively explored in microbial cells. For assessing the biocompatibility of QDs for bioimaging applications, the solubility, QD uptake and toxicity of the QDs are the primary prerequisites. Following the uptake by the cell, size dependent routing within the cells facilitates the QDs in reaching the cell organelles whereas metal ions remain sequestered within the cytoplasm^47^. QDs are also known to significantly affect the cellular redox balance.

However, confocal images (Fig. 6C) shows cellular uptake of QDs after incubation for 4 and 6 hrs and clear blue fluorescence visible inside the fungal cells indicates the internalization of the QDs. We tried our best to eliminate the probability that “non-washed QDs could remain in the solution after washing procedures” by giving stringent washing twice to remove the unlabelled QDs. The cells were then viewed under Confocal microscope and the blue fluorescence was observed only inside the cells (Fig. 6C, fluorescence and DIC images) and not from the regions outside the cell envelope. In case, there were any non-internalized QDs remaining in solution, it would also be visible under the microscope outside the cells but no fluorescence was observed outside the cells which could pertain to the non-washed QDs in solution. We checked the fluorescence of QDs in suspension and it was clearly different from QDs internalized in the cells (Fig. S6 (Supplementary Information)).

The results of confocal images were further validated by monitoring the fluorescence intensity using spectrofluorimeter. The same cell suspension was used for both the confocal imaging and spectrofluorimetric assays. The uptake of QDs by *Candida* cells were analyzed by measuring the relative fluorescence intensity. The cells without QDs were maintained as blank separately to nullify the auto-fluorescence. Figure 6B depicts the mean R_f_ values ± the standard deviation (SD) of three sets of independent experiments, R_f_ Intensity was calculated by subtracting the fluorescence intensity for cells incubated without QDs (blank) from fluorescence intensity for cells incubated with AIS and AIS@ZnS QDs respectively.

It has been shown by others that the thio-capped CdTe QDs could be well internalized into living cells *in vitro* over a time period of about 1 hr, since surface of the thio-capped QDs contain carboxylic groups, which may function as the biological interfacing^30,48^.

### ROS studies

**Figure 7:**
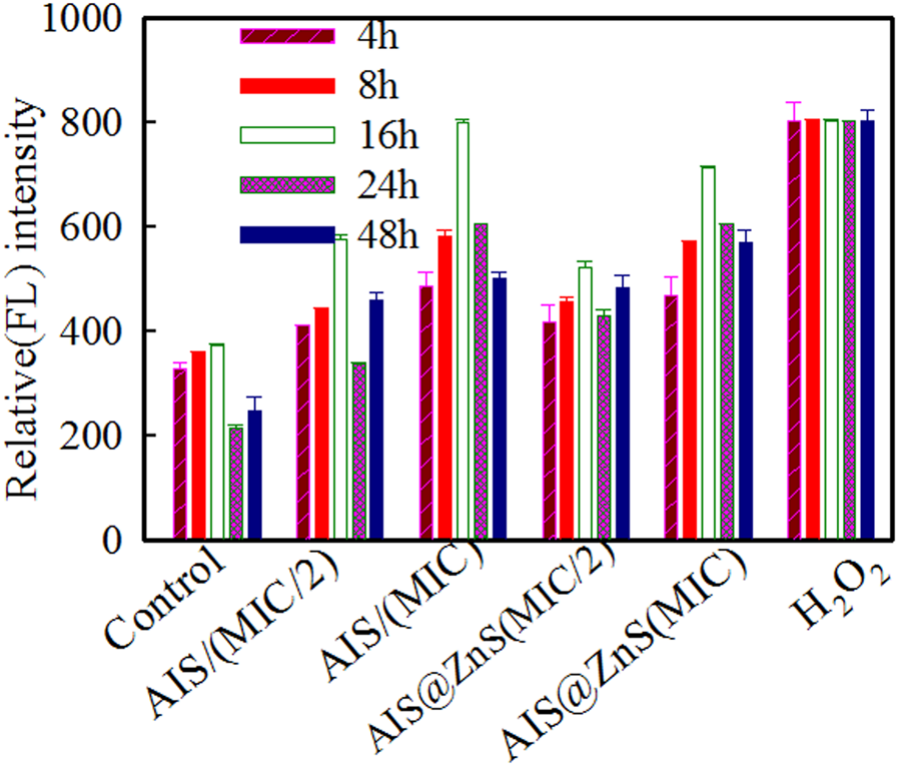
Evaluation of intracellular ROS produced in the *Candida* cells in the presence of sub-inhibitory and toxic concentrations of AIS and AIS@ZnS respectively. ROS production was estimated by relative fluorescence (Rf) intensity values and Y axis denotes the mean Rf values ± the standard deviation (SD) of three sets of independent experiments, Rf Intensity was calculated by subtracting the fluorescence intensity for cells incubated without DCFDA (blank) from fluorescence intensity for cells incubated with DCFDA.

**Figure 8:**
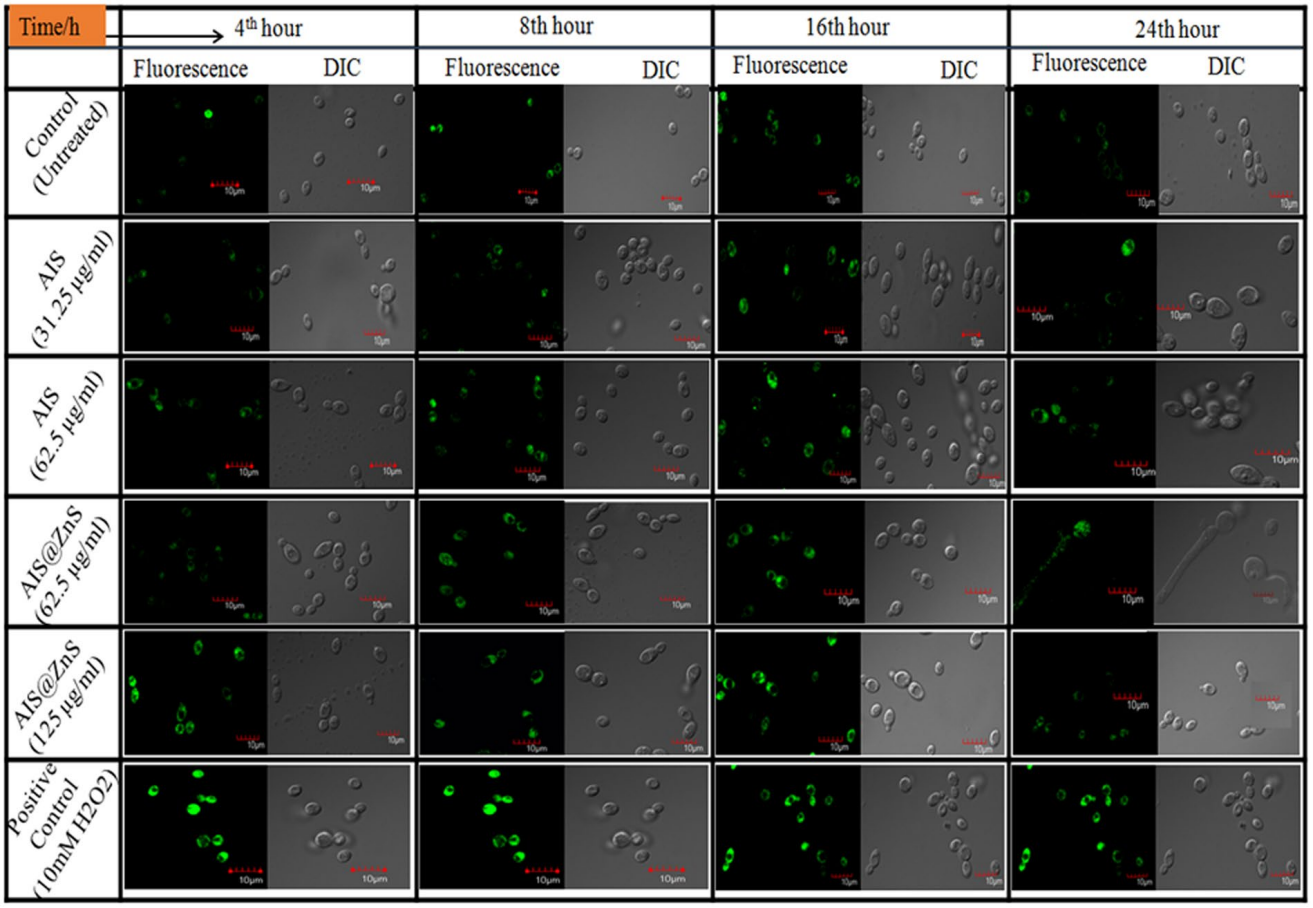
Confocal images showing green fluorescence for time dependent intracellular ROS produced in fungal cells in response to QDs. Endogenous ROS was assessed in *Candida* cells grown in the absence (Control) and presence of 31.25 µg/ml (sub-inhibitory) and 62.5 µg/ml (MIC_90_) of AIS and also in presence of 62.5 µg/ml (sub-inhibitory) and 125 µg/ml (MIC_90_) of AIS@ZnS respectively at different time intervals (4 hr, 8 hr, 16 hr and 24 hr). Each left panel of different time intervals depict the fluorescence image (higher fluorescence indicates higher ROS production) taken in a Confocal microscope and each right panel for different time intervals represents the DIC image at magnification 100X. The obtained results were compared with Control (Untreated) and positive control (cells treated with 10 mM hydrogen peroxide, H_2_O_2_).

Role of reactive oxygen species (ROS) is emerging among the mechanisms proposed for QD induced toxicity and action of several nanomaterials have been associated with augmented ROS levels. Needless to mention, the nature of surface modifications is likely to play an important role in toxicity and uptake. Therefore, we examined the intracellular ROS generated in the fungal cells in response to the internalized QDs after incubation for 4, 8, 16, 24 and 48 hrs. The fluorescent probe, DCFDA measures hydroxyl, peroxyl and other reactive oxygen species activity within the cells and provides an ultrasensitive fluorometric one step assay. The advantage of fluorometric method is the higher sensitivity (Pico molar range) in comparison to other methods like colorimetric assay etc. Therefore, it now is offered as an attractive alternative to the other methods^49^ and is used for detecting ROS in microbial pathogens. Alkaline hydrolysis of DCFDA converts it to stable 2′, 7′- dichlorofluorescein (DCFH) which is stable for few hours but is converted to fluorescent DCF upon oxidation and the intensity of fluorescence observed is due to intracellular accumulation of ROS produced by equal number of cells. Toxic (MIC_90_ i.e. the concentration required to inhibit 90% of the cells) and sub-inhibitory (half of the concentration required to inhibit 90% of the cells) concentration of QDs were used for assessing the intracellular ROS levels. Intracellular ROS production in the fungal cells grown in absence and presence of 31.25 µg/ml (sub-inhibitory) and 62.5 µg/ml (MIC_90_) of AIS and 62.5 µg/ml (sub-inhibitory) and 125 µg/ml (MIC_90_) of AIS@ZnS respectively was estimated at different time intervals as described in Materials and Methods. At all the time points, the ROS generated was higher at MIC_90_ as compared to the sub-inhibitory concentrations of QDs (Fig. 7). Incubation for 8 hrs at respective sub-inhibitory concentrations of AIS and AIS@ZnS appears to the most suitable time point for bioimaging experiments, when the cells are in log phase (exponentially growing) and the ROS generated within the cells are comparable to the basal level of intracellular ROS generated in absence of QDs (control). Confocal images in Fig. 8 show time dependent intracellular ROS generation in fungal cells in response to QDs leading to a conclusion that corroborates with the data obtained for ROS generation through fluorescence measurements.

Although, the uptake of AIS@ZnS was better than AIS after incubation for 8 hrs, both the QDs show significantly good cellular uptake at this time point. To summarize, sub-inhibitory concentration of AIS and AIS@ZnS appear to have lower ROS production and therefore, may be used to optimize the use of QDs as biostable fluorescent markers in microbial live cell imaging. To make these QDs more appropriate for targeted cellular imaging and other biological applications, they would require to be conjugated to molecules which will not alter the functions of QDs.

## Conclusions

We have developed a facile approach for the synthesis of monodispersed highly fluorescent AIS and AIS@ZnS quantum dots. These quantum dots have been synthesized directly in aqueous medium using glutathione as capping agent. Indium content plays a dominating role in tuning the emission intensity, color and bandgap of these quantum dots. These AIS@ZnS quantum dots exhibit very high quantum yield of 49% corresponding to Ag:In molar ratio of 1:4. Furthermore, AIS@ZnS was found to be more effective and biocompatible than AIS QDs and hence, these can be efficiently used for bioimaging applications. High quantum efficiency of the synthesized particles was utilized to probe *Candida albicans* cells using confocal microscopy. We present the potential of highly fluorescent AIS particles for use as probes for molecular and cellular imaging in *Candida albicans*. Our studies, thus offer unique solutions for the future development of safe, fluorescent probes for clinical applications in live cell imaging and drug delivery. The novelty in biological applications of these aqueous dispersible core-shell quantum dots is because of their long fluorescence lifetimes (>300 ns), very low cytotoxicity, great optical and long colloidal stability in the physiological pH range. Long fluorescence lifetimes will help to overcome the problem of photobleaching encountered frequently during the use of other fluorescence labels and will thus, allow the monitoring of live systems for extended periods of time.

Our results demonstrate that the shelling of the core QDs improves the photo-stability and photodynamic efficacy in our samples. We have further estimated and provided a comparison of the production of singlet oxygen to emphasize on the mechanism of phototoxicity. The concerned QDs can be used as vehicles for delivery of photosensitizers for cancer diagnosis and photodynamic therapy (PDT) applications. It is evident from our results that these stable, monodispersed core and core-shell structures show promising potential for photochemotherapy of cancer. It is generally believed that singlet oxygen mechanism dominates during PDT and its presence is responsible for the cytotoxic species produced by phthalocyanine photosensitizers^50^. The generation of singlet oxygen is significantly improved by shelling the core QDs with ZnS shell.

## Electronic supplementary material

Supplementary Information
