# Supplementary material for: Bandgap Tunable AgInS based Quantum Dots for High Contrast Cell Imaging with Enhanced Photodynamic and Antifungal Applications
Source: Sci Rep. 2018 Jun 19;8:9322. doi: 10.1038/s41598-018-27246-y (PMC6008435; doi:10.1038/s41598-018-27246-y)
Supplement: Supplementary file 1 — Supplementary Information [file 41598_2018_27246_MOESM1_ESM.pdf]

Bandgap Tunable AgInS based Quantum Dots for High Contrast Cell Imaging with Enhanced Photodynamic and Antifungal Applications

Irshad Ahmad Mir[1], Radhakrishanan VS[2], Kamla Rawat[3,4], Tulika Prasad[2,3*] and H. B. Bohidar[1,3*]

[1]School of Physical Sciences, Jawaharlal Nehru University, New Delhi, India

[2]Advanced Instrument Research Facility, Jawaharlal Nehru University, New Delhi, India

[3]Special Centre for Nano Sciences, Jawaharlal Nehru University, New Delhi, India

[4]Inter University Accelerator Centre, New Delhi, India

*Corresponding authors email: bohi0700@mail.jnu.ac.in, prasadtulika@hotmail.com

Tel: +91 11 26704637, Fax: +91 11 2674 1837

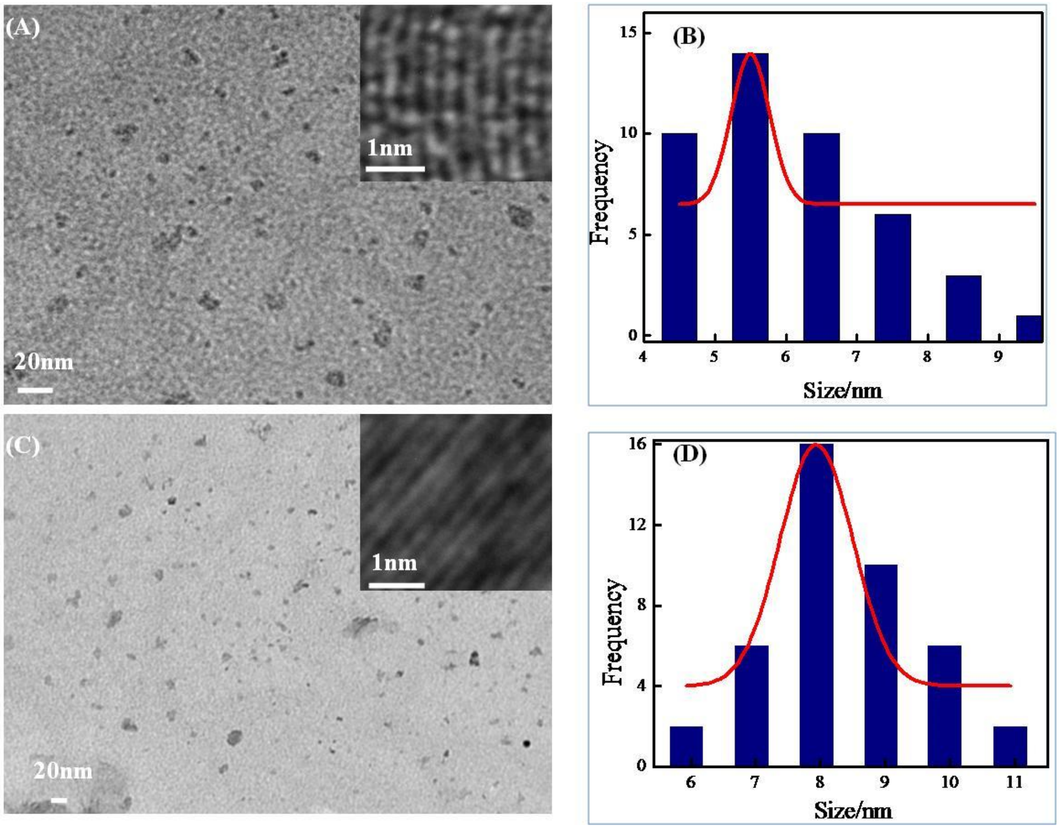

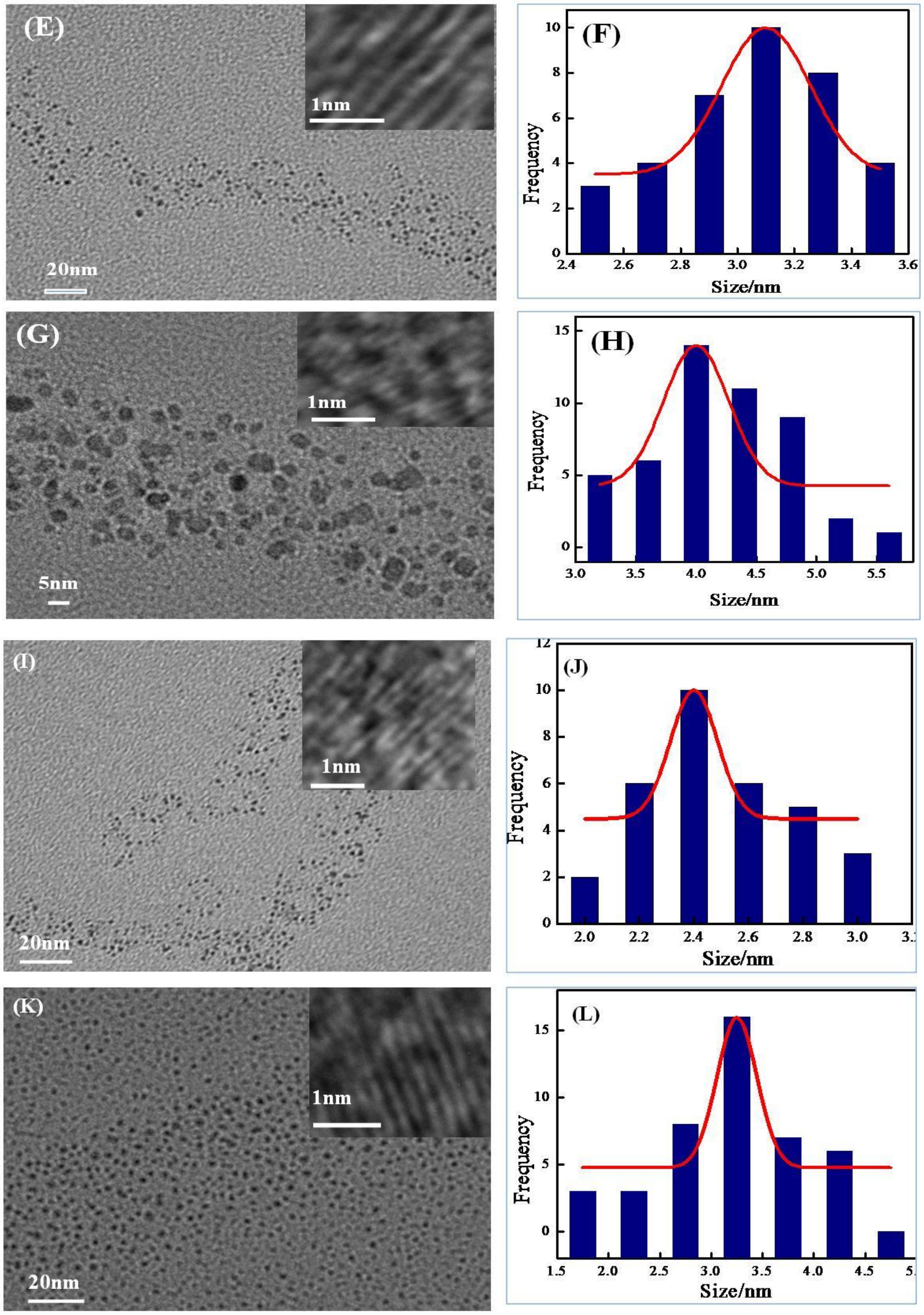
(E)
1nm
20nm
(F)
Frequency
Size/nm
(G)
1nm
5nm
(H)
Frequency
Size/nm
(I)
1nm
20nm
(J)
Frequency
Size/nm
(K)
1nm
20nm
(L)
Frequency
Size/nm

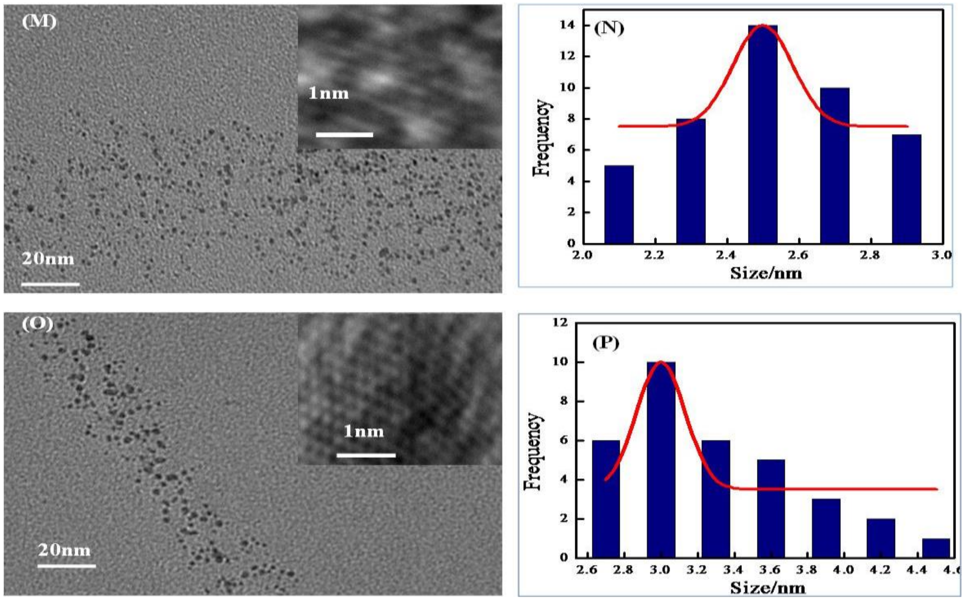

Figure S1. TEM (inset HRTEM) images and TEM histogram of AIS core only and AIS@ZnS core-shell QDs for different Ag:In ratio{1:1(**A** and **C**), 1:2.5 (**E** and **G**), 1:5 (**I** and **K**), 1:9 (**M** and **O**)}. Note 1:4 (Ag:In) ratio is shown in figure (**2**).

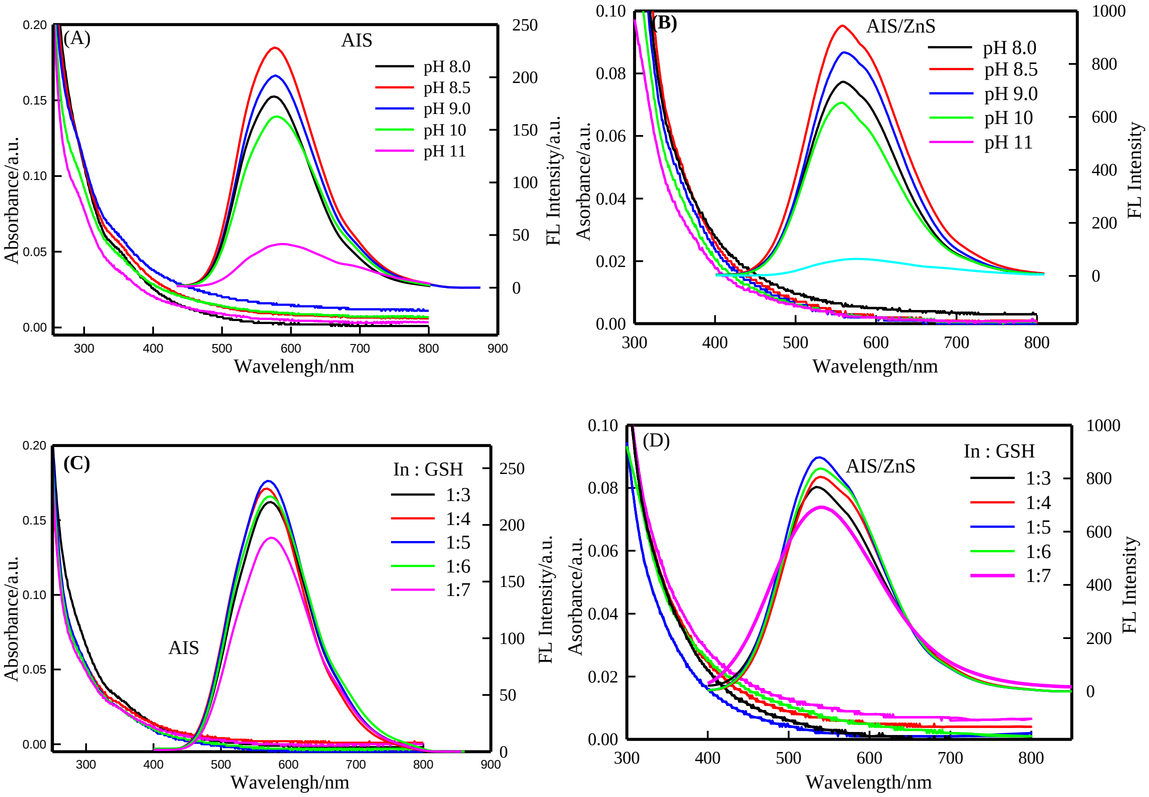

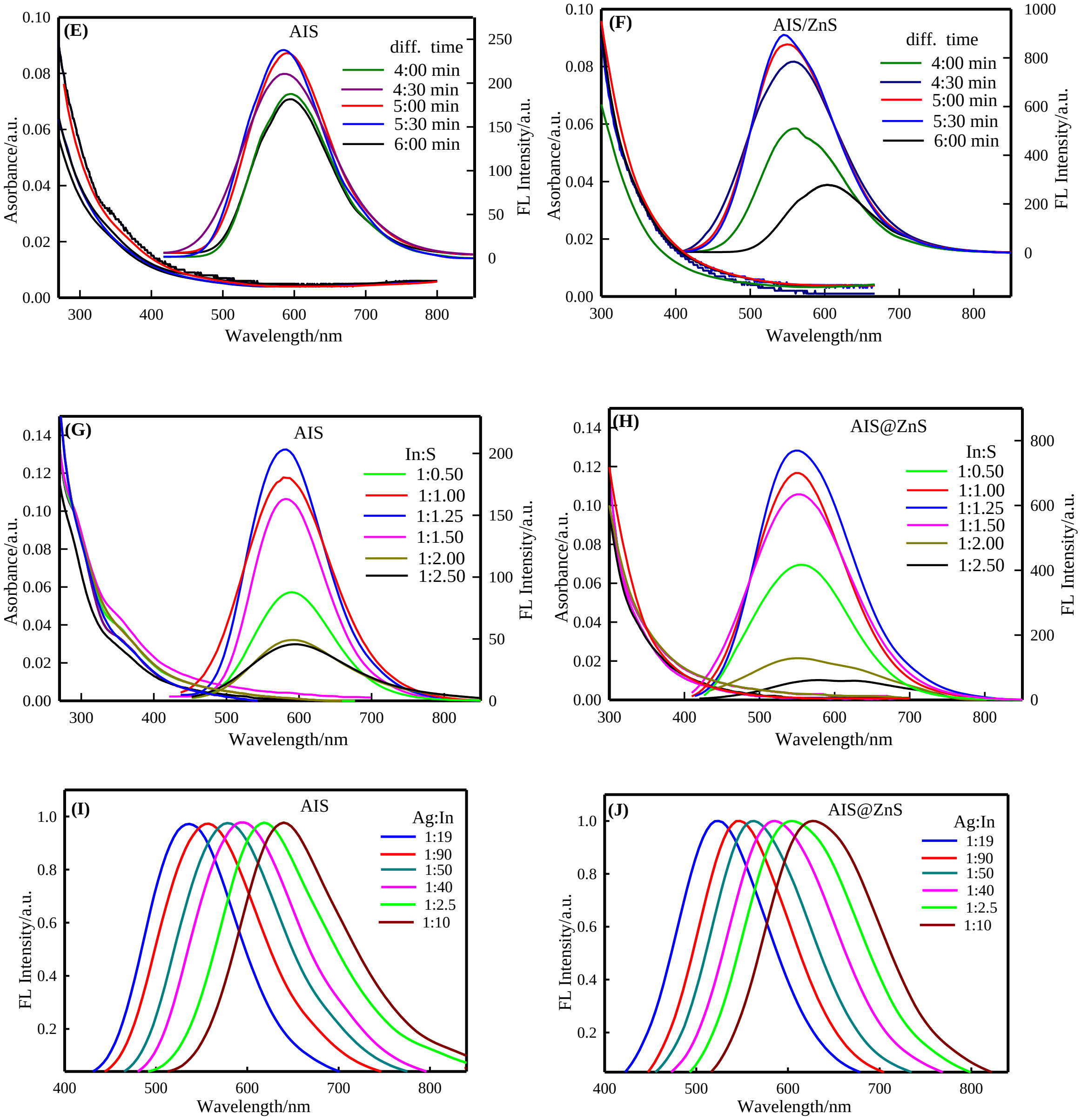

Figure S2. (**A**) and (**B**) Absorbance and fluorescence spectra at different pH respectively for AIS core only and AIS@ZnS core-shell QDs. (**C**) and (**D**) Absorbance and fluorescence spectra at different In:GSH ratio respectively for AIS core only and AIS@ZnS core-shell QDs. (**E**) and (**F**) Absorbance and fluorescence spectra at different irradiation time respectively for AIS core only and AIS@ZnS core-shell QDs. (**G**) and (**H**) Absorbance and fluorescence spectra at different In:S ratio respectively for AIS core only and AIS@ZnS core-shell QDs.(**I**) and (**J**) normalized fluorescence spectra at different Ag:In ratio respectively for AIS core only and AIS@ZnS core-shell QDs.

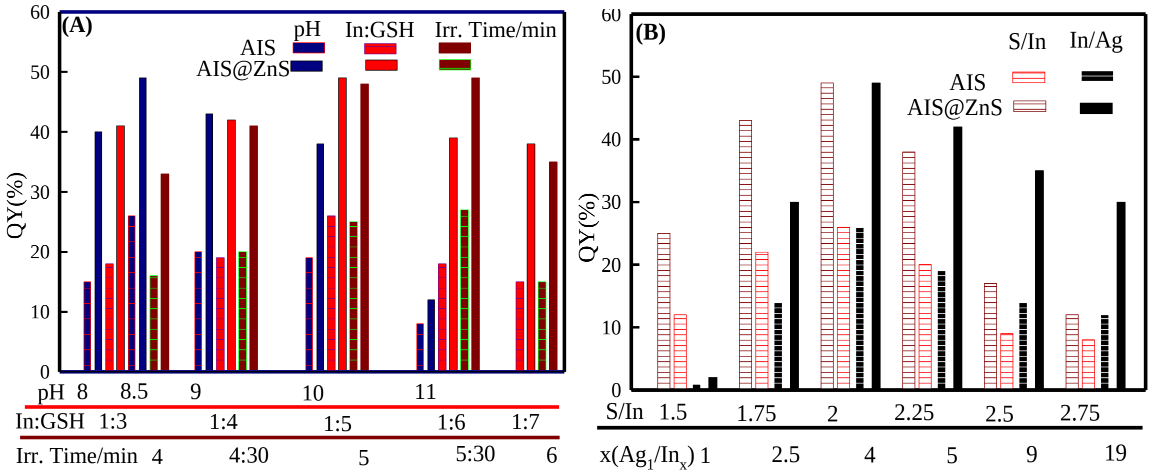

Figure S3. Quantum yield optimization ((**A**) at different pH, In:GSH and irrd. time and (**B**) S:In and In:Ag) for AIS core only and AIS@ZnS core-shell QDs.

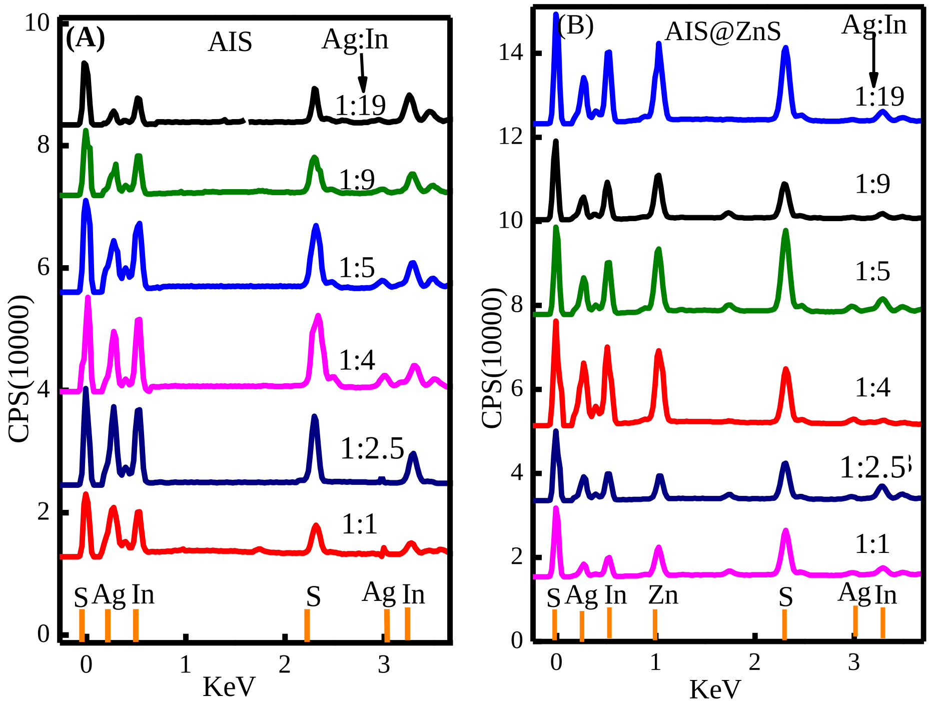

Figure S4. EDX of (**A**) AIS core only and (**B**) AIS@ZnS core-shell QDs (for different In:Ag) which conforms the existence of Ag, In, S and Zn in the synthesized quantum dots.

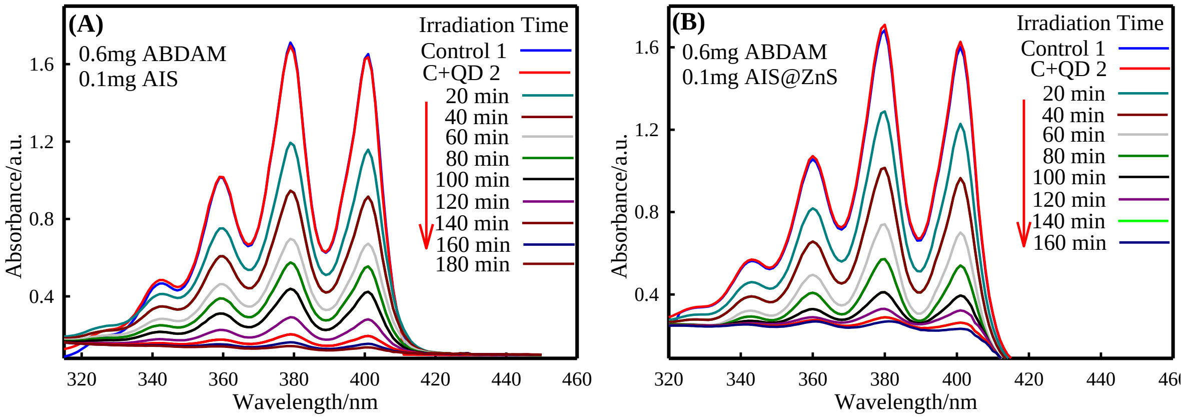

Figure S5. Time dependent changes in ABMDMA spectrum upon UV illumination in the absence and presence of AIS and AIS@ZnS quantum dots.

Fluorescent QDs **Merge**

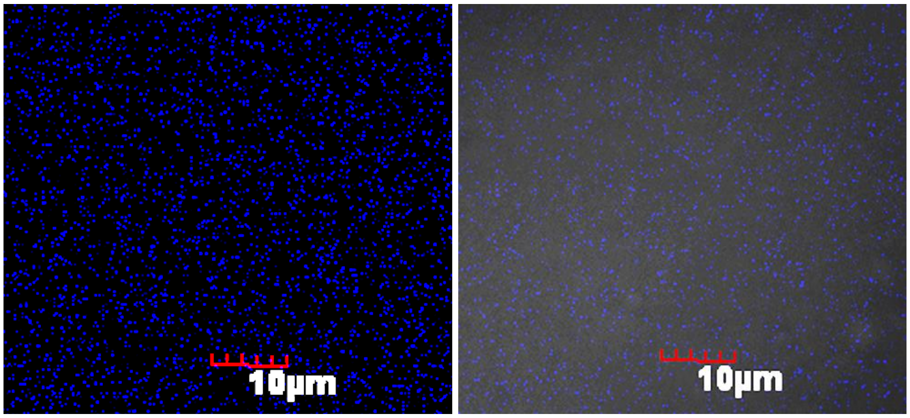

Figure S6. Left panel shows fluorescence of QDs in suspension under Confocal microscope and right panel indicates the merge for the phase-contrast micrographs and fluorescence images at magnification 100X.

Table S1. Elemental analysis of AIS and AIS@ZnS core-shell quantum dots for different cation ratio.

**Feed molar Ag:In**	**AIS EDX weight %**				**AIS@ZnS EDX weight %**			
	**Ag**	**In**	**S**	**Zn**	**Ag**	**In**	**S**	**Zn**
1:1	29±2	26±2	43±2	N	06±1	06±0.5	55±3	31±2
1:2.3	23±2	30±1	46±2	N	21±1	28±2	36±2	14±1
1:4	14±1	49±1.5	35±2	N	07±0.5	29±2	37±2	25±1
1:5	14±1	43±2	41±3	N	05±0.5	28±2	51±3	14±1

1:9	12±1	52±3	35±1	N	08±1	27±2	51±2	13±1
1:19	4±0.5	62±2	32±1	N	03±0.4	37±1	49±3	09±0.5

Here N means neither used in precursor nor detected from EDX. In this cation range, sulfur and silver precursor are kept same in the entire range.

Table S2. Amplitude constants ratio and the decay time obtained from TRFS data.

Sample	τ_1/ns	A (%)	τ_2/ns	B (%)	Life time/ns
AIS	439	65	118	35	326
AIS@ZnS	561	71	128	31	438
